# A Clinical Study to Evaluate the Safety and Efficacy of Oral Administration of Microscopic Dose Gold Nanoparticle (AuNP) on Knee Joint Health and Function in Arthritis Patients

**DOI:** 10.3390/jfmk7030052

**Published:** 2022-06-22

**Authors:** Xuesheng Han, Edlynne Avelar, Amber Mathai, David Vollmer, Richard Lehman

**Affiliations:** 1 4Life Research, Scientific Research Division, Sandy, UT 84070, USA; davidv@4life.com; 2 Professional Athletic Orthopedics, St. Louis, MO 63122, USA; edlynnea93@gmail.com (E.A.); mathai.amber@gmail.com (A.M.)

**Keywords:** gold nanoparticle (AuNP), knee joint health, rheumatoid arthritis, osteoarthritis, arthritis

## Abstract

The purpose of this clinical study was to determine whether gold nanoparticle (AuNP) supplementation at a dosage of 0.34 mg elemental gold per day can improve knee joint health, function, and quality of life for arthritis patients. A total of 51 participants (24 male and 27 female, age 62.1 ± 13.1) were followed for 20 weeks through a three-phase longitudinal study. Both subjective and objective parameters were used to measure changes in joint health and function, as well as quality of life. The study found patients’ Knee injury and Osteoarthritis Outcome Score (KOOS) improved with statistical significance. It was reported that 71.42% of the cohort experienced improvements in their perceived knee pain and 61.22% with improvements in knee stiffness. Majority of objective measurements such as pain with range of motion and specific exercises requiring proper knee health and function did not show statistically significant improvement but did show a positive improving trend in support of AuNP supplement. Study cohort showed statistically significant improvements in two specific exercises: sit-to-stand and single-leg squat. By the end of the study, 70% of the study cohort indicated that they would continue to take the supplement even after the study concluded. Though the study has limitations and is not definitely conclusive, it was the first clinical study to show that oral micro-dosage of AuNP as low as 0.34 mg daily is safe and effective for both rheumatoid arthritis and osteoarthritis patients. This study opened way for the use of AuNP in both clinical and daily settings to improve joint health and function for both average and athletic users.

## 1. Introduction

In 2006, the Centers for Disease Control and Prevention (CDC) estimated 20% of the population experienced knee joint discomfort [1]. Since the CDC’s survey, the numbers have increased, and joint health continues to play a large role in the activities of daily living and contributes to burdening one’s quality of life with age [2]. Pain, inflammation, mobility, and flexibility all contribute to joint discomfort, and can be triggered by one’s lifestyle. For young, adolescent, and adult athletes, physical demand can lead to injuries such as meniscal and ligament tears, or it can exacerbate wear-and-tear of the joints [3,4]. For sedentary lifestyles and occupations, the lack of exercise causes joints to become stiff, weak, and inflexible, increasing the risk of injury during above-average activities [5]. Furthermore, in the labor force, repetitive motions can cause microtraumas in the joints, especially with weighted tasks such as squatting and bending, resulting in injuries, early onset arthritis, and chronic joint pain [6]. Thus, means of supporting knee joint health and comfort continues to be important, as is finding alternatives to nonsteroidal anti-inflammatory drugs (NSAIDs) and steroidal treatments.

Gold-based therapy has a long history of medical use in treating rheumatoid arthritis (RA). Jacques Forestier demonstrated in 1929 that gold salt injections relieve joint pain in patients suffering from rheumatoid arthritis and sometimes lead to complete remission [7]. Thereafter, gold salts therapy had been commonly used until the 1990s, after which presumably less toxic and more efficient treatments developed. 

Some studies showed that compared to gold salts, AuNP is a more potent and effective anti-arthritic agent and has significantly less toxicity [1,8,9]. Specifically, in a small exploratory clinical study published in 1997, ten patients with severe rheumatoid arthritis who failed previous treatments saw drastic improvements in their knee swelling and pain when placed on a colloidal metallic gold treatment for 6 months [10]. Furthermore, it was noted that the participants experienced no observable toxicity. It has also been demonstrated in subsequent studies that the cytotoxic effects of AuNP are nearly zero or at least significantly less than other nanoparticles such as silver nanoparticles [11].

Though the mechanism of action is not fully understood, it has been shown that AuNP, primarily via its potent anti-inflammatory and antioxidant properties, works by invading macrophages to prevent their infiltration and stimulation of inflammation around the joints, without killing the immune cells [12]. 

In a recent open-label study with 63 osteoarthritis (OA) patients, intra-articular gold injection improved their Knee injury and Osteoarthritis Outcome Score (KOOS) and Western Ontario and McMaster Universities Osteoarthritis Index (WOMAC) scores, with no reported severe adverse effects (SAE) [13]. In another newly published exploratory clinical study, gold microparticles injected intra-articularly in osteoarthritic knee joints relieved perceived pain and generated an inflammation modulatory effect based on proteome changes found in synovial fluid SF and serum [14].

In this study, AuNP was given as an oral liquid supplement called Gold Factor (GF) to arthritis (RA and OA) patients. The study rationale partially followed a previous trial performed by Dr. Richard Lehman, orthopedic surgeon, around 2010. That trial (unpublished data) showed that a similar gold supplement improved joint health and function in osteoarthritic patients. It also found that taking the supplement at 3 oz a day for 14 days followed by 2 oz a day for another 14 days had the most significant results in overall joint health and function and decrease in joint and muscle pain. 

The purpose of this clinical study was to evaluate whether AuNP supplement at microscopic dosage given in daily treatments could improve joint health and function, as well as quality of life as measured by both subjective and objective parameters. 

## 2. Methods

This study was reviewed and approved by an ethics committee before its commencement (Ethic Committee Name: 4Life Research Ethic Committee; Approval Code: GF-AuKHC46; Approval Date: 16 Janurary 2020). The study was registered on ClinicalTrials.gov (NCT05347602). The study started on 3 August 2020 and ended on 30 December 2020.

A total of 60 subjects were screened for eligibility into the study. Participants were initially screened through phone calls, followed by a physical exam screening. Selected participants were fully educated about the risks and benefits of their participation and autonomies, and signed informed consent was acquired before proceeding. Participants understood they could leave the study at any point. X-ray examinations were completed for each potential subject and their affected knee(s). Screenings were aimed at excluding fractures, injuries that required immediate surgical intervention, and/or end stage degenerative arthritis. When selecting participants the following inclusion criteria were utilized: the participants were between 18 and 87 years of age; they had arthritis; they were suffering from chronic knee pain; they failed treatments to date including surgery, physical therapy, and NSAIDs; the participant had to agree to maintain the same eating, exercise, and sleep arrangements for the whole duration of the study. When selecting participants, the following exclusion criteria were utilized: having bacterial, intra-articular, or knee infections; having any knee-related partial or complete total knee replacement within 3 months prior to the start of the study; having a recent (3 months prior to start of the study) knee injection; currently on NSAIDs; currently pregnant or lactating. 

Fifty-one (51) subjects (24 male and 27 female, age 62.1 ± 13.1) were determined eligible for the study after the screenings and then were divided into two groups, AuNP Supplement (*n* = 26) and Placebo (*n* = 25) and were followed for a total of 20 weeks (5 months). The study was partially double-blinded and divided into three phases as follows:

Phase 1 (8 weeks): Supplement and Placebo groups ingested 3 oz of Supplement and Placebo per day, respectively, for the first four weeks as a loading dose and then switched to 2 oz per day for weeks 5–8. This phase was double-blinded.

Phase 2 (4 weeks): Both groups underwent a month-long “wash out” period, during which they did not ingest any of the Supplement or Placebo. This lasted from weeks 9–12. This phase was not double-blinded as all participants and the researchers were aware of the wash-out procedure. 

Phase 3 (8 weeks): Both groups took the Supplement at 3 oz per day as a loading dose for four weeks (weeks 13–16), and then 2 oz per day for the final four weeks (weeks 17–20). This phase was open-labeled, as all participants and the researchers were aware that all participants were ingesting the Supplement. 

All subjective and objective measurements were taken at four timepoints: before the start of Phase 1 (referred to as T0) and at the end of each phase (referred to as T1, T2, and T3, consecutively). 

Subjective measurements were collected through KOOS survey. The KOOS survey was chosen due to its repeatability, and it gave scores of 0–100 for five separate categories: Symptoms, Pain, Function & Daily Living, Sports & Recreational Activities, and Quality of Life. Under KOOS scoring guidelines, a score of zero represents extreme knee problems, and 100 represents complete function of the knee with no pain, discomfort, or other joint-related problems. Furthermore, office surveys were filled in by all participants at all four timepoints T0, T1, T2 and T3.

Objective measurements were collected by the physician and two registered nurses who conducted physical examinations of each subject. C-reactive protein (CRP) levels were measured for all study participants at four timepoints. Participants also completed a physical therapy assessment with a registered physical therapist. Nine participants (four in the Supplement group and five in the Placebo group) were selected randomly for an in-depth DorsaVi assessment in addition to the physical therapy undertaken by all participants. 

During the whole study period, all participants refrained from taking pain medication, NSAIDs, or other treatments aimed at resolving joint pain and/or inflammation. Subjects took the recommended doses once daily, at the same time each day. They did not take the supplement within 30 min of any food or drink. Each subject was given detailed instructions in a booklet that provided dates for when to switch from 3 oz to 2 oz, as well as dates that notified transitions into the next phase. The booklets also allowed space for observations to be written down regarding participant knee joint health and function, and any other pertinent changes to overall health during each phase. 

## 3. Materials

AuNP supplement was provided by the study sponsor, 4Life Research, LLC (Sandy, UT, USA). Safety profiles and toxicology experiments were conducted at Toxi-Coop Zrt. Test Facility in Budapest, Hungary, prior to this human clinical study. It was reported that GF did not cause any SAEs in tested male and female Han:WIST rats following a consecutive 60-day oral treatment. Clinical observations, functional observations, body weight development, food consumption and feed efficiency, clinical pathology, and organ pathology were all tested, and resulted in a No Observed Adverse Effect Level (NOAEL) of 10 mg/kg body weight/day. Potential mutagenic activity of GF was examined in the bone marrow of male NMRI mice. This micronucleus test found that there were no biologically or statistically significant increases in the frequency of micronucleated polychromatic erythrocytes in the treated mice with the maximum NOAEL daily dose, nor did the supplement show any genotoxic activity. Lastly, in a study using Chinese Hamster lung V79 cells, GF did not induce structural chromosome aberrations, under the NOAEL maximum daily dose, and was thus classified as non-clastogenic [15].

The AuNP used in the study was an aqueous suspension of clean-surfaced, faceted gold nanoparticles that have extraordinary catalytic capabilities [16]. The AuNP were 8–28 nm in diameter, with varying shapes from bipyramids to polyhedra. To avoid contamination, the AuNP were suspended in ultra-pure water and were not coated with any proteins or molecules to avoid negative reactions within living tissues. This resulted in a rose-pink color due to the incident light inducing a specific resonance, known as the localized surface plasmon resonance. The AuNP supplement used in this study contains elemental gold at 6 ppm. Study dosage was 2 oz per day, which is about 0.34 mg elemental gold per day. 

For the Placebo, water and red dye were mixed to create a placebo liquid with the same rose-pink shade as the AuNP supplement, along with flavoring that matched the AuNP supplement.

### Data Analysis

Statistical analysis was conducted using R, Version 4.0.3. Comparison between Supplement and Placebo groups were analyzed by using two-sample *t*-tests, and only a *p* value ≤ 0.05 is considered statistically significant.

The T0 to T3 comparisons were beginning of the study to the end. In this case, the supplement group had followed supplement, wash-out, and supplement; and the placebo group had followed placebo, wash-out, and supplement.

The “first use” group combines Phase 1 for the supplement group with Phase 3 for the placebo group to get a larger sample of pre-post comparisons but without a comparison group.

## 4. Results

### 4.1. KOOS Scores

**Figure 1:**
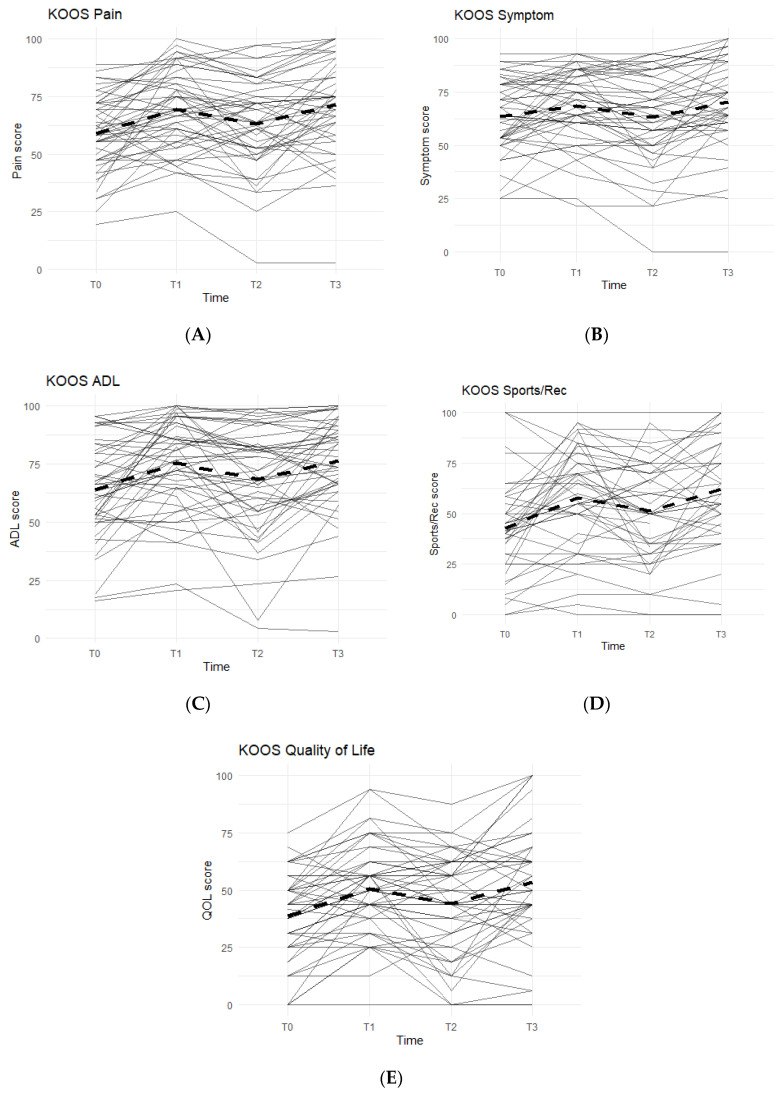
KOOS Scores of the Entire Study Cohort (*n* = 51). Score averages were graphed for the entire cohort throughout each phase. Solid lines represent individual participant scores. Thick dashed lines represent group averages. (**A**) shows the KOOS Pain scores from T0–T3. (**B**) shows the KOOS Symptom scores from T0–T3. (**C**) shows the KOOS ADL scores from T0–T3. (**D**) shows the KOOS Sports/Rec scores from T0–T3. (**E**) shows the KOOS Quality of Life scores from T0–T3.

The KOOS survey is divided into five categories: Pain, Symptoms, Function in Activities of Daily Living (ADL), Function in Sports and Recreation, and Quality of Life. An improvement between time points was indicated by an increase in KOOS scores. In all KOOS categories, patterns followed what were anticipated: increase in KOOS scores during Phase 1, decrease during Phase 2 (washout), and a final increase during Phase 3 (Figure 1). 

Data reported no statistically significant differences between the Placebo and Supplement Groups after Phase 1 (*p* > 0.05 for all KOOS categories).

The difference between Supplement vs. Placebo Groups for each category during Phase 2 (T1–T2) was not statistically significant but displayed a general trend favoring the Supplement’s effect on perceived knee joint health and function.

The final phase (Supplement, T2 to T3) produced KOOS score improvements that were statistically significant. From T2–T3, about 74% of the total participants (*n* = 51) saw an increase in KOOS Pain Scores, and there was an average 8.19 points increase in scores. In the Symptoms category, 70.8% marked improvement with an average increase of 7.90 points. In the ADL category, the data reported an average increase of 8.53 points and 70.8% of the participants receiving higher scores. The Sports and Recreational section showed an average point increase of 9.76, with 65.9% of the participants experiencing improvements in their scores. Lastly, there was a 9.11 average point increase for 58.3% of the entire cohort in the QOL section. 

Overall improvements were analyzed for each KOOS category comparing T0 to T3 for the whole cohort (*n* = 51), and all improvements in every KOOS category were statistically significant. In addition, majority of participants reported improvements: 77.1% improved in their Pain scores, 61.2% improved their Symptoms scores, 79.6% improved in their ADL scores, 74.4% improved in their Sports and Recreation scores, and 73.5% improved in their QOL scores. 

To further increase the study’s statistical power, the data from the Supplement Group’s first use of Supplement during Phase 1 (T0–T1) and the data from the Placebo Group’s first use of Supplement during Phase 3 (T2–T3) were combined to form the First Use of Supplement cohort. This provided a data set with *n* = 51 that analyzed the first 8 weeks on First Use of Supplement. All KOOS category scores showed a statistically significant improvement in the cohort (*p* < 0.01). The results of KOOS for the First Use of Supplement Cohort were summarized in Table 1. 

### 4.2. In-Office Surveys

In-office surveys were conducted at T0 to gauge the baseline level of Pain and Stiffness in the affected knee(s). Surveys at T1, T2, and T3 focused on perceived changes in the knee(s) due to the Supplement or Placebo intake. A five-option scale was used to track changes: Significantly Worse, Slightly Worse, No Change, Slightly Better, and Significantly Better.

After Phase 1 (T0–T1), 44% of the Placebo Group and 44% of the Supplement Group reported their Pain felt “Better.” For Knee Stiffness, 40% of the Placebo Group reported doing “Better” compared to 52% of the Supplement Group. However, the difference between the two groups by the end of Phase 1 was not statistically significant. At the end of the study (T0 to T3), 71.42% of all participants experienced improvement of their Knee Pain, and 61.22% experienced improvement of their Knee Stiffness.

To increase the study’s statistical power, the data of each phase when a group was taking the supplement were combined: T0–T1 for Supplement Group, T2–T3 for all participants. This gave *n* = 77. These results were then compared to that of each phase when a group was not on the supplement: T0–T1 for Placebo Group, T1–T2 for all participants. This gave *n* = 76.

This analysis showed that 68.5% of the participants (37% Slightly Better and 31.5% Significantly Better) experienced improvement in their Knee Pain. Furthermore, when not taking the supplement, a majority of participants reported No Change in knee pain (48.6%) or some level of worsening knee pain (23%). The differences between Supplement vs. No Supplement phases provided statistical significance (*p* < 0.001) in Knee Pain improvement when taking the supplement.

### 4.3. CRP Levels

C-Reactive protein levels were measured at T0 for baseline scores, and then measured again at T1, T2, and T3. As CRP levels are biomarkers of systemic inflammation, it was expected to see a decrease in CRP levels when taking AuNP supplement. 

Interestingly, after Phase 1 (T0–T1), the Placebo Group experienced an average decrease of 2.48 mg/L in CRP levels, while the Supplement Group experienced an average increase of 0.08 mg/L. During Phase 2 (T1–T2), as expected, 64% of the participants in both groups experienced an average increase in CRP levels of about 0.5 mg/L. Then, during Phase 3 (T2–T3), 50% of all participants experienced an average decrease of 0.58 mg/L in their CRP levels.

At the end of the study (T0–T3), there was an average decrease of 1.31 mg/L, with a total of 54.2% of the participants experiencing an improvement in their CRP levels. However, these changes were not statistically significant, but they do show a trend of decreasing CRP levels when taking AuNP supplement.

### 4.4. Physical Therapy

From T0–T3, all participants completed a physical assessment conducted by an assigned physical therapist. None of the recorded data showed a statistically significant difference between the Supplement and Placebo groups during Phase 1. The following are comparisons of T0 data from all participants with their final T3 data.

**Figure 2:**
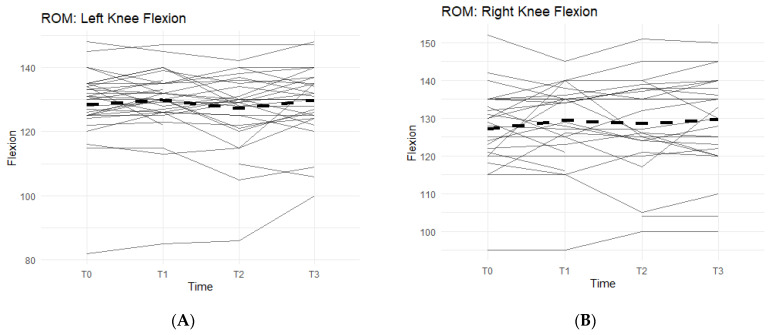

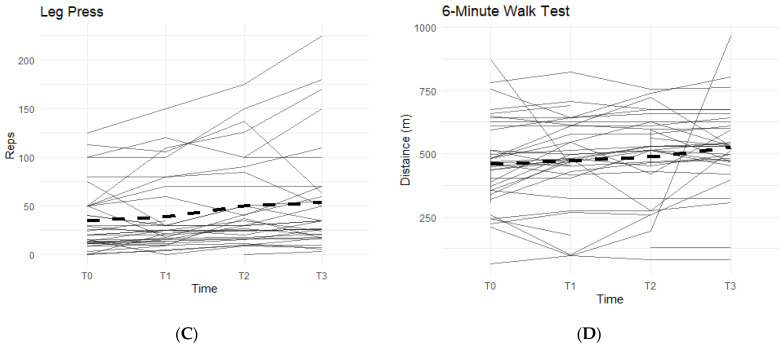
Bilateral Flexion, Leg Press, and Walk Test Changes Over Time During the Study Period. The figures show the changes in flexion of the left and right knees separately, and the changes in leg press repetitions and distance covered in a six-minute treadmill walk. Only the results for the affected knees were included in each figure. Solid lines represent individual participant scores. Thick dashed lines represent group averages. (**A**) shows left knee flexion from T0–T3. (**B**) shows right knee flexion from T0–T3. (**C**) shows leg press reps from T0–T3. (**D**) shows the 6-min walk test from T0–T3.

Of the measurements, the Range of Motion (ROM) extension of the left and right knees and bilateral quad circumferences did not experience a statistically significant change from T0 to T3 (Figure 2A,B). 

Bilateral knee flexion showed a positive trend suggesting a correlation between the Supplement and increase in flexion ROM. Comparing T0 measurements of the left knee to T3, 50% of participants saw an increase in their flexion (Figure 2A). On average, there was 1.73 ± 5.98 degrees increase in left knee flexion experienced by the entire cohort. The right knee flexion pattern is similar to the left knee. From T0 to T3 measurements, 61.5% of participants saw an increase in their flexion ROM (Figure 2B). There was an average increase of 2.77 ± 8 degrees.

Furthermore, there was also a pattern that displayed an average increase in completed repetitions of leg presses, and an increase in distance covered during a six-minute walk on a treadmill while on a daily regimen of the supplement. From T0 to T3, 81.8% of participants experienced an increase in completed leg press repetitions (Figure 2C). On average, the participants were able to complete up to 17–18 more reps of leg presses (±35.24). Although not statistically significant, there is an observable increase in repetitions while taking the supplement. A similar result is seen in the six-minute walk test, with an average increase in distance of 30.25 m (±172.25 m) during T0 to T3, and a total of 78.8% of participants being able to walk a further distance on the treadmill (Figure 2D). 

**Figure 3:**
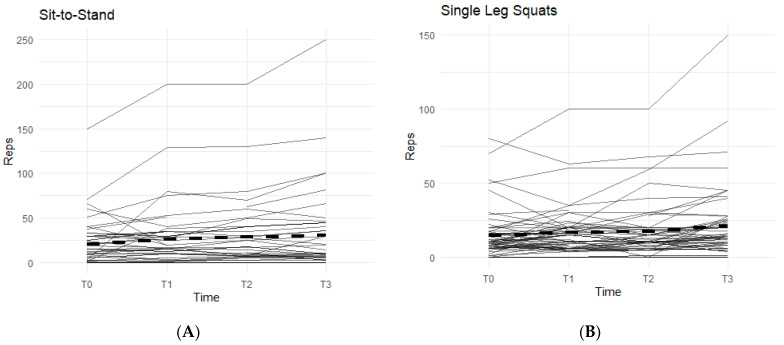
Sit-to Stand and Single-Leg Squats Changes Over Time During the Study Period. The figures show the changes in total repetitions of sit-to-stand squats and single-leg squats. Only the results for the affected knees were included. Solid lines represent individual participant scores. Thick dashed lines represent group averages. (**A**) shows reps of sit-to-stand squats from T0–T3. (**B**) shows reps of single-leg squats from T0–T3.

The other two exercises, sit-to-stand squat and single-leg squat, were measured in each affected leg separately. The data from both exercises displayed an increasing pattern when on the supplement, and both were statistically significant (Figure 3).

In the sit-to-stand category, 52.3% of the participants were able to increase their repetitions of sit-to-stand squats when taking the supplement (Figure 3A). On average, participants were able to complete 11.36 ± 26.22 more repetitions by T3 than their T0 baseline count. These results were statistically significant (95% CI: 3.39–19.34, *p* < 0.01). 

For single-leg squats, 58.9% of the participants were able to increase their completed repetitions while on supplement (Figure 3B). On average, participants increased their repetitions by 7.29 (SD 14.60). Both outcomes were statistically significant (95% CI: 3.38–11.19, *p* < 0.01), showing a positive correlation with the supplement. 

### 4.5. Physical Exams

From T0–T3, all participants were given a physical knee exam by the physician or nurse practitioners. During the exams, most observations and specialized tests were performed for left and right knees separately for participants who indicated bilateral knee pain. Otherwise, only one knee is accounted for each participant. Gait, stance alignment, patellar grinding, and patellar tracking were analyzed as one observation per participant.

For all analyzed variables, changes from T0 to T1 were compared between Supplement and Placebo groups using a multilevel regression model with terms for group, time, and their interaction. The Group Supplement estimate represents how the Supplement Group differs from the Placebo Group across the whole study. This includes baseline measurement and therefore is not a proper measurement of the overall effect from the supplement. The TimeT1 variable represents how scores at T1 differ from T0 across all participants, but it does not differentiate between the Supplement and Placebo Groups. The interaction term, indicated as Group Supplement × TimeT1 in the tables below, represents the estimated effect of the supplement over the placebo over the first phase. This is the best “effect” estimate.

**Figure 4:**
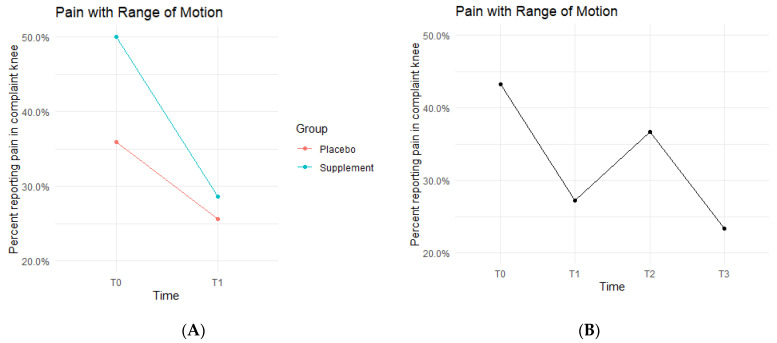
Pain with ROM Changes Over Time. Average percentages of participants experiencing pain with ROM were graphed on a plot. (**A**) compares pain with ROM changes in the Supplement vs. Placebo group from T0 to T1. (**B**) shows averages in pain with ROM for the entire cohort as a whole at times T0, T1, T2, and T3. Overall, the cohort started with 43.2% experiencing pain with ROM, and dropped to 23.4% by the end of the study. Only affected knees were included.

Pain with ROM rates decreased in both the Placebo and Supplement Groups from T0-T1 (Figure 4A). The Placebo Group dropped from 35.9% of the participants experiencing pain with ROM at T0 to only 25.6% at T1. This improvement rate is most likely a result of the placebo effect. The Supplement Group dropped from 50% to 28.6%, which is a greater improvement rate than the Placebo Group. The cohort pain with ROM then increased from T1–T2, with 27.2% experiencing pain with ROM at the start of Phase 2 compared to 36.7% by the end of Phase 2. Lastly, from T2–T3, the cohort was at a final rate of 23.4% participants with ROM pain (Figure 4B).

The fixed effects from the multilevel models of pain with ROM at times T0 to T1 showed the Supplement Group showed a larger decrease in pain with ROM (i.e., higher rates of improvement when on the supplement) (Table 2). The differences between Supplement vs. Placebo Groups were not statistically significant but showed a trend in favor of the supplement. 

**Figure 5:**
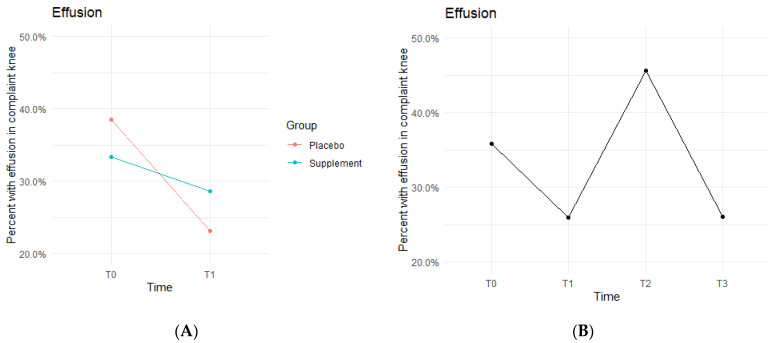
Effusion Changes Over Time. Average percentages of participants experiencing effusion were graphed on a plot. (**A**) compares Supplement vs. Placebo from T0 to T1. (**B**) shows averages for the entire cohort as a whole at times T0, T1, T2, and T3. Overall, the cohort started with 35.8% experiencing knee effusion, and dropped to 26% by the end of the study. Only affected knees were included in the data.

Knee effusion for affected knees in both the Placebo and Supplement Groups experienced a decrease from T0 to T1 (Figure 5A). The Placebo Group dropped effusion rates from 38.5% of participants to 23.1%, whereas the Supplement Group went from 33.3% to 28.6%. There was then an increase from T1 to T2, with the entire cohort starting Phase 2 with 25.9% experiencing knee effusion and ending the phase with 45.6% knee effusion rates. There was a final sharp decrease from T2 at 45.6% to T3 at 26% (Figure 5B). This aligned with the hypothesized result of a decrease in overall effusion rates.

The fixed effects from the multilevel models of normal gait at times T0 to T1 for the Supplement Group showed higher rates of effusion improvement when on the supplement (Table 3). The differences between Supplement vs. Placebo Groups were not statistically significant but showed a trend in favor of the supplement.

**Figure 6:**
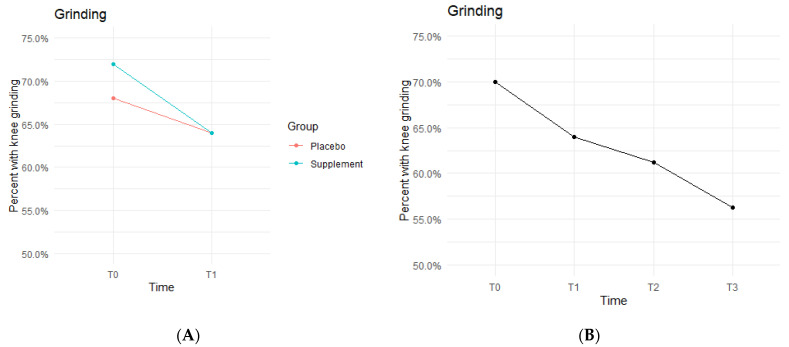
Knee Grinding Changes Over Time. Average percentages of participants experiencing grinding were graphed on a plot. (**A**) compares Supplement vs. Placebo from T0 to T1. (**B**) shows averages for the entire cohort as a whole at times T0, T1, T2, and T3. Only affected knees were included in the data.

The Placebo and Supplement Groups experienced an overall decrease in percentage of participants with patellar grinding from T0 vs. T3 (Figure 6A). At the start of the study 70% of the cohort experienced some knee grinding, and by the end, rates dropped to 56.2% (Figure 6B). This aligns with the hypothesized outcome of grinding percentages decreasing by the end of the study.

The fixed effects from the multilevel models of grinding at times T0 to T1 showed the Supplement Group showed a larger decrease in probability of knee grinding when on the supplement (Table 4). The differences between Supplement vs. Placebo Groups were not statistically significant but showed a trend in favor of the supplement.

Patellar tracking, defined as the patella shifting out of its central groove during knee extension and/or flexion, was observed by the physician or nurse practitioners during each physical exam.

**Figure 7:**
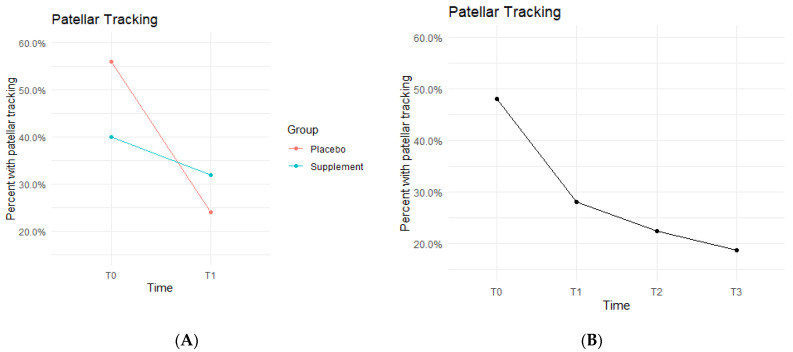
Patellar Tracking Changes Over Time. Average percentages of participants with poor patellar tracking were graphed on a plot. (**A**) compares Supplement vs. Placebo from T0 to T1. (**B**) shows averages for the entire cohort as a whole at times T0, T1, T2, and T3. Only affected knees were included in the data.

Both Placebo and Supplement Groups experienced an overall decrease in percentage of participants with patellar tracking from T0 to T3 (Figure 7A). From T0 to T1, the Placebo Group dropped from 14 participants with patellar tracking down to 6 participants, whereas the Supplement Group only decreased from 10 to 8 knees.

By the end of the study (T0–T3), the overall patellar tracking rates dropped from 24 total knees to 9 knees. This was not statistically significant (Figure 7B) but did show a trend in favor of the supplement. The fixed effects from the multilevel model of patellar tracking (Table 5) at times T0 to T1 showed the Supplement Group had a larger decrease in probability of knee-tracking when on the supplement. 

**Figure 8:**
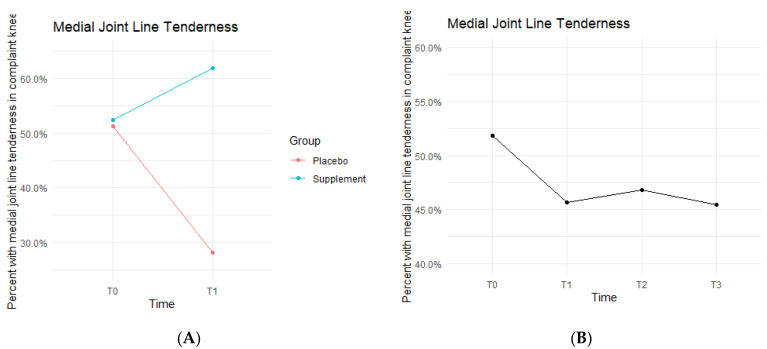
MJL Tenderness Changes Over Time. Average percentages of participants with MJL tenderness were graphed on a plot. (**A**) compares Supplement vs. Placebo from T0 to T1. (**B**) shows averages for the entire cohort as a whole at times T0, T1, T2, and T3. Only affected knees were included in the data.

Medial joint line (MJL) tenderness data showed the Placebo and Supplement Groups experienced contrasting results throughout each phase of the study (Figure 8). From T0 to T1, the Placebo Group decreased in MJL tenderness rates, whereas the Supplement Group increased. Then, from T1 to T3, the Placebo Group experienced an increase in rates while the Supplement Group decreased. Overall, rates of MJL tenderness started at 51.9% at T0 and dropped to 45.5% by T3.

**Figure 9:**
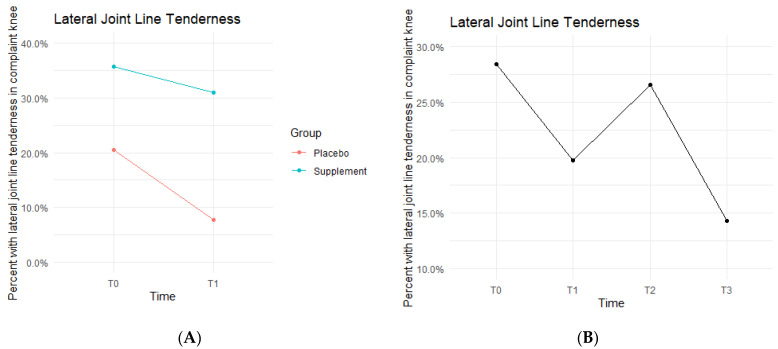
LJL Tenderness Changes Over Time. Average percentages of participants with LJL tenderness were graphed on a plot. (**A**) compares Supplement vs. Placebo from T0 to T1. (**B**) shows averages for the entire cohort as a whole at times T0, T1, T2, and T3. Only affected knees were included in the data.

Lateral joint line (LJL) tenderness rates also decreased from T0 to T1, with the Placebo Group starting the study at 20.5% exhibiting LJL tenderness and dropping to 7.7% by the end of Phase 1. The Supplement Group saw a decrease from 35.7% to 31% (Figure 9A). There was then an increase experienced by the entire cohort from T1 to T2, starting at 19.8% and increasing to 26.6%. Then, there was a final decrease from T2 to T3 with rates lowering to 14.3% by the end of the study (Figure 9B). There was no statistical difference between the Placebo and Supplement Groups. However, the overall decrease in LJL tenderness showed a trend in favor of the supplement.

The Placebo and Supplement Groups were also tested for posture/stance alignment, crepitus, and specialized tests that serve as an indicator of instability or damage of the meniscus (McMurray’s test) and the ACL (Pivot Shift and Anterior Drawer tests). By the end of the study, it was concluded that there was no statistically significant difference between the Placebo and Supplement Groups, nor was there a statistically significant improvement comparing T0 to T3. These tests also did not reveal a trend in favor of the supplement or placebo.

### 4.6. DorsaVi Assessments

DorsaVi assessments used sensor patches to calculate in-depth balance and movement measurements during four knee-loading exercises. Ten (10) out of all participants were chosen at random to participate in a DorsaVi assessment with CORA Physical Therapy in addition to the normal physical therapy exercise assessments. Out of the ten participants, five were in the Placebo Group and five were in the Supplement Group. One participant dropped out of the study, leaving four in the Supplement Group. Therefore, the analysis was done on data from nine participants (four in Supplement and five in Placebo). The analyses below include exercise total scores and, where relevant, the exercise speed measurements. The speed measurements were suggested by the physical therapist as an important indicator of knee strength, balance, and control.

**Figure 10:**
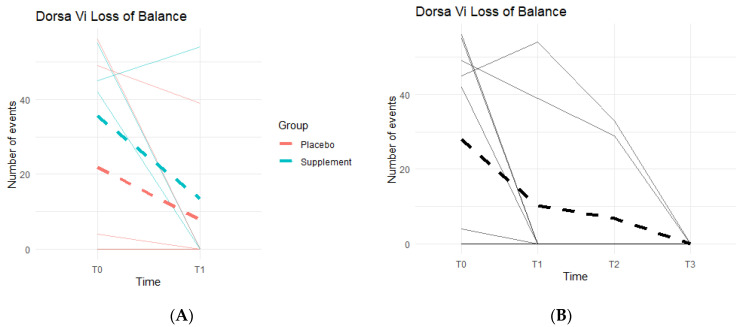
LOB Changes Over Time. DorsaVi LOB events were graphed on a plot. (**A**) compares Supplement vs. Placebo results from T0 to T1. (**B**) shows averages for the entire cohort as a whole at times T0, T1, T2, and T3.

Loss of Balance (LOB) Events provide a better insight into knee favoritism and stability vs. instability. As knee symptoms increase in number and in severity, there is an expected increase in LOB events. Both groups experienced a decline in LOB events (Figure 10A), meaning patients improved their balance throughout the study. From T0 to T1, three out of the five people in the Placebo Group improved, with an average decrease of 14 LOB events, and two out of the four in the Supplement Group improved with an average decrease of 22 LOB events. From T1–T2, one person from the Placebo Group and one from the Supplement Group improved. The same results were seen from T2–T3. The change in the DorsaVi population, from T0–T3, was an average decrease of six LOB events (Figure 10B). Six participants out of nine improved.

**Figure 11:**
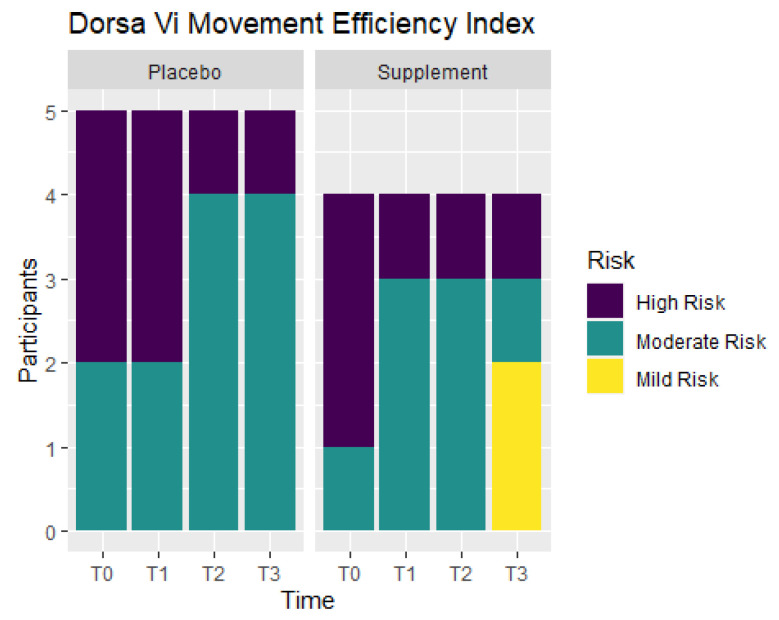
Movement Efficiency Changes Over Time. DorsaVi Movement Efficiency in the Supplement and Placebo Groups were recorded to compare results at points T0, T1, T2 and T3. Results show the majority of the participants improved by T3. High Risk participants are in purple, Moderate Risk in green, and Mild Risk in yellow.

Movement efficiency indices were recorded throughout the study to determine levels of risks for sports/movement injuries. Participants ranked at No Risk, Mild Risk, Moderate Risk, or High Risk. Figure 11 shows the number of participants in each ranking at T0, T1, T2, and T3. From T0 to T1, the Placebo Group did not show any improvement, but the Supplement Group experienced a change of three participants in the High Risk category improving to the Moderate Risk category. At T0, there were three participants ranked at Moderate Risk and six ranked at High Risk. By T3, there were two participants that improved to Mild Risk status, five participants that were Moderate Risk, and only two that remained at High Risk. In total, four out of nine participants improved their movement efficiency from T0 to T3.

Squats provided insight on knee strength and stability by measuring endurance, lumbar flexion (i.e., placing more weight towards the heels or the toes when squatting down), and lumbar lateral shift (i.e., placing more weight towards the left or right side of the body). A total of 20 squat repetitions were completed per assessment. It was expected to see an increase in scores as well as lumbar flexion and lateral shifts nearing or reaching 0 degrees.

From T0 to T1, both the Placebo and Supplement Groups had one participant improve their overall squat score, thus there was no statistical significance that favored the supplement during Phase 1. Contrary to the expected results, there was an overall decline in scores from T0 to T3, with only one participant that improved their scores.

**Figure 12:**
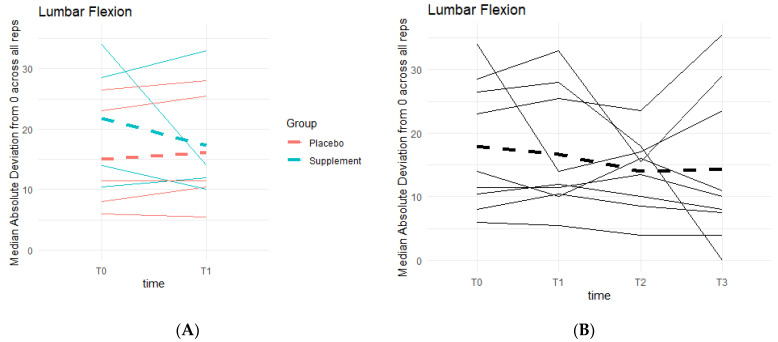
Squat Lumbar Flexion Deviation Changes. Lumbar flexion deviations were recorded. The median absolute deviations from 0 degrees across all repetitions as a function of time were then graphed. (**A**) compares Supplement vs. Placebo Groups from T0 to T1. (**B**) shows averages for the entire cohort at T0, T1, T2 and T3.

Weak knee function can cause improper squat form and balance, resulting in deviations from a centered 0 degrees of lumbar flexion. There was a general pattern of deviations decreasing as participant averages neared 0 degrees (Figure 12). During Phase 1, the differences between the Placebo and Supplement groups were not statistically significant. Comparing T0 to T3, eight out of the nine participants in the cohort improved their scores, and although not statistically significant, it showed a pattern in favor of the supplement’s effects on knee health and function.

**Figure 13:**
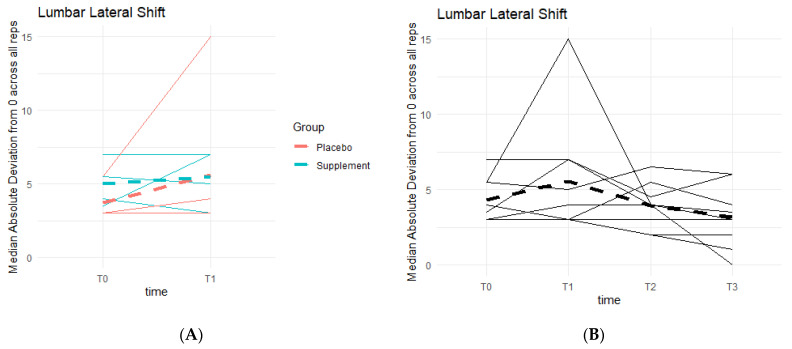
Squat Lateral Shift Deviations. Lateral shift deviations were recorded. The median absolute deviations from 0 degrees across all repetitions as a function of time were then graphed. (**A**) compares Supplement vs. Placebo Groups from T0 to T1. (**B**) shows averages for the entire cohort at T0, T1, T2 and T3.

Similar to lumbar flexion, the goal was to reach 0 degrees of lumbar lateral shift, indicating a stable squat position from beginning to end, without unevenly overloading the left or right leg based on pain and/or favoritism. There is a general pattern of deviations decreasing as participants neared 0 degrees (Figure 13). Phase 1 revealed no statistically significant differences between the two groups. Comparing T0 to T3, six out of the nine participants in the cohort improved their scores, providing a pattern in favor of the supplement’s effects on knee health and function.

Single-leg squats rely on knee strength, mobility, and health. The DorsaVi assessments represent knee capabilities by the speed at which the participants lower into and out of the single-leg squat, and the tibial inclination reached during each repetition. The slower the single-leg squat was completed and the higher the tibial inclination, the more eccentric control and strength the participant had in his/her movement. The speed goal was for participants to reach less than or equal to 20 degrees per second per repetition and a tibial inclination greater than or equal to 30 degrees. Below the targeted goal meant participants were unable to perform a full single-leg squat, an indication of symptomatic knee(s).

**Figure 14:**
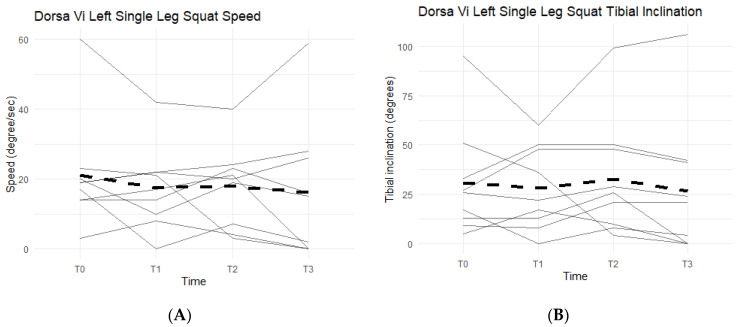
Left Single-Leg Squat Changes Over Time. Changes in single-leg squat speed and tibial inclination were graphed to compare results at T0, T1, T2, and T3. (**A**) graphs speed measured in degrees per second as a function of time. (**B**) graphs tibial inclination in degrees as a function of time. Solid lines represent individual data, dashed lines are group averages.

Neither left leg squat scores, speed, nor tibial inclination differences between the Placebo and Supplement Groups after Phase 1 were statistically significant. Furthermore, only two participants in the entire cohort improved their scores from T0 to T3, which was also statistically insignificant (*p* > 0.05). Figure 14A shows six out of the nine participants (66.7%) in the cohort had decreased their speed by the end of the study. On average the participants saw a decrease of 4.78 degrees per second (±10.69 degrees). Tibial inclination data (Figure 14B) showed that, from T0 to T3, five out of the nine participants (55.6%) increased their tibial inclination. Although the results in scores, speed, and tibial inclination were not statistically significant, they showed a trend in favor of Supplement in improving knee joint health and function.

**Figure 15:**
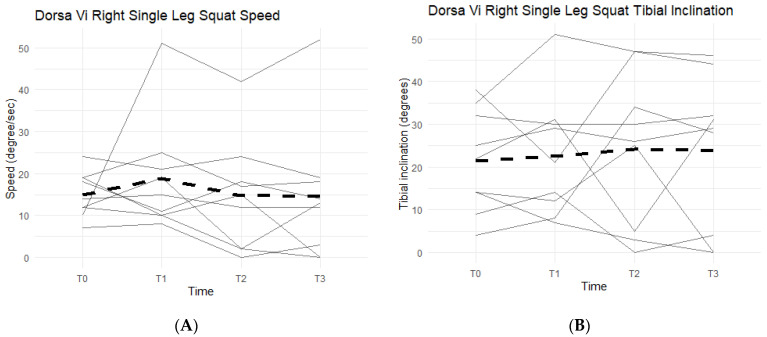
Right Single-Leg Squat Changes Over Time. Changes in single-leg squat scores, speed, and tibial inclination were graphed to compare results at T0, T1, T2, and T3. (**A**) graphs speed measured in degrees per second as a function of time. (**B**) graphs tibial inclination in degrees as a function of time. Solid lines represent individual data, dashed lines are group averages.

Right single-leg squat scores, speed, and tibial inclination showed no statistically significant difference between the Placebo and Supplement groups at the end of Phase 1. From T0 to T3, two participants increased their scores, seven decreased their speed, and three increased their tibial inclination (Figure 15). Although not statistically significant, the speed data showed support in favor of Supplement and its aid in improving knee joint health and function.

Lastly, single-leg hops, single-leg hop plants, and ankle lunges were performed, with total scores, speeds, and tibial inclinations recorded. The data for these exercises were inconclusive and showed no statistically significant differences between the Placebo and Supplement Groups at the end of Phase 1, or improvements by the end of the study.

### 4.7. Acceptability

Acceptability of AuNP supplement was analyzed at the end of the study (T3). Participants were asked if they would continue taking the product after the study in their final exit survey. Out of 48 participants that answered the question, 70% answered a definitive “Yes” that they would continue taking the product.

## 5. Discussion

To the best of our knowledge, this study is the first clinical trial to evaluate the efficacy and safety of oral administration of AuNP at micro-dosage on joint health and function in arthritis patients. The present study did not show statistically significant differences between Supplement and Placebo groups likely due to insufficient statistical power. However, in-group comparison analyses showed that AuNP supplement significantly improved several aspects of joint health and function in those arthritis patients.

Numerous studies of AuNP have been conducted in in vitro and preclinical rheumatoid arthritis (RA) models. Koushki and colleagues have published a recent review on the multifaceted roles of AuNP in RA [12] with a focus on its anti-inflammatory and antioxidant effects. Existing studies highlighted the great potential of AuNP as a candidate for RA treatment and warranted the need to evaluate how much AuNP may exert their anti-arthritic effects. The present clinical study using micro-dosage of AuNP was the first clinical study of its kind and provided valuable knowledge to answer such questions.

In a small exploratory clinical study, ten patients with severe rheumatoid arthritis who failed previous treatments orally took 30 mg of colloidal gold daily and thereafter weekly for 4 weeks and monthly for an additional 5 months. At the end of the 6-month study, these 10 patients saw drastic improvements in their knee swelling and pain and no toxicity or adverse effects were found [10]. The present study administered AuNP orally to both OA and RA patients at micro-dosage 0.34 mg elemental gold per day, which is much lower compared to 30 mg per day used in the abovementioned study. Nevertheless, similar results were observed in the present study: participants saw improvements in their perceived knee pain, comfort, and function.

In a recent open-label study with 63 OA patients, intra-articular AuNP injection improved their KOOS and WOMAC scores [13]. No SAE was reported. Another clinical study showed that intra-articular gold microparticles injection in knee osteoarthritis joints relieved perceived pain of OA patients [14]. The results from these two clinical studies in OA patients are consistent with the results from the present study in both RA and OA patients. Oral administration has advantages over knee injection such as better safety and compliance.

These further suggested that oral administration of micro-dosage AuNP as low as 0.34 mg daily is safe and effective for both RA and OA patients.

To the best of our knowledge, the present study was also the first clinical study to employ objective measurements to evaluate the efficacy of AuNP oral administration on arthritis patients. The present study did not find statistically significant improvement in the majority of those objective measurements in the AuNP Supplement Group compared to Placebo group, largely due to lack of statistical power. However, the measurements identified a trend towards better improvement in the Supplement group. It is important to note that these observed improving trends in the physical assessments and exercises are not attributed to the knee(s) and muscles adapting to the movements. Participants did not engage in any strenuous activity outside of their normal routines, nor practice any of the exercises outside of the assessments. Thus, there was enough time between each phase such that the knees did not adapt and improve based on repetition and habit.

Further assessment into the seemingly non-significant improvements in objective measurements suggested that they are largely consistent with and supportive of the observed improvements in subjective measurements. Pain with ROM started at a baseline rate of 43.2% of total knees in the cohort and dropped down to 23.4% by the end of the study. Knee effusion dropped from 35.8% to 26% in the population, knee grinding from 70% to 56.2%, patellar tracking from 48% to 18.8%, and lateral joint line tenderness from 28.4% to 14.3%. These symptomatic objective measurements suggested that the AuNP supplement was able to aid in the mobility of the knees and increase the comfort felt from any twisting, pivoting, flexion, or extension, and thus at least partially explained the improvements in perceived knee pain, comfort, and function for arthritis patients. Further, as the general environment of a knee joint is improved, it is expected for the knees to experience enhanced capabilities when performing exercises. This was observed during the physical assessments with physical therapists, and with the DorsaVi assessments conducted for the nine participants in the study. In the sit-to-stand exercise, 47.6% of the participants were able to improve and increase their total repetitions completed after the first two months of taking AuNP supplement. This was found to be statistically significant (*p* = 0.04). Single-leg squats during physical assessments were also improved by 50.9% of the cohort (statistically significant; *p* < 0.01). These were the only two exercises that were enhanced in a statistically significant manner.

From the DorsaVi assessments, arguably the most important exercise that involves the knees is the basic squat. Results showed that eight out of the nine participants improved their lumbar flexion (i.e., moving forwards towards the toes or backwards towards the heels) and six out of nine improved their lateral shift (i.e., placing more weight on the left or right leg). Single-leg squats from the DorsaVi assessments were also improved throughout the study. The speed at which single-leg squats are performed may indicate how well a knee can withstand the weight load during the movement. The higher the speed, the less control and less ability the knee has, and the lower the speed, the more in-control the participant is in executing the squat. Tibial inclinations are also an important factor, as they tell how deep into the single-leg squat the participant can reach: the greater the inclination, the deeper the squat, the stronger the knee. The present study showed that six out of nine participants decreased their left single-leg squat speed, seven out of nine decreased their right single-leg squat speed, and five out of nine increased their left single-leg squat tibial inclination. These objective improvements, though non-significant statistically, are supportive of the subjective improvements in knee comfort and function reported by arthritis patients.

It was hypothesized that the CRP levels would decrease when taking AuNP supplement. CRP levels in a healthy person should remain below 5 mg/L, but are elevated when a person is fighting a bacterial or viral infection. Elevated CRP levels have also been attributed to certain autoimmune diseases, such as RA. CRP can elevate within 6 h, and has a half-life of around 19 h in the body [17]. Without an active stimulus in the body to increase CRP production and release, these levels can decrease and remain low. Majority of the participants in the study cohort held average levels of CRP (at or under 5 mg/L). However, there was a recorded decreasing pattern in CRP levels from T0 to T3 in 54.2% of the cohort. The average decrease was 1.31 mg/L. It was also observed that 64% of participants experienced an increase in average CRP levels during the washout phase from T1 to T2 when they were not on AuNP supplement. These results support the hypothesis of AuNP helping to decrease and maintain relatively lower CRP levels, demonstrating the anti-inflammatory effect of AuNP supplement in arthritis patients. 

The results of serum CRP levels in the present study are consistent with another recent clinical study in OA patients conducted by Rasmussen et al. in 2022. Intra-articular gold microparticle injection in knee osteoarthritis joints saw 28 different proteins were downregulated and 11 upregulated (*p* < 0.05) mainly associated immune response and inflammation regulation in patients’ synovial fluid (SF). Similarly, 31 proteins were downregulated and 1 was upregulated in patients’ serum (*p* < 0.05), reflecting key immune response, inflammation, and anatomical structure development processes. Based on these proteomic analyses, the authors concluded that intra-articular gold microparticles injection in knee osteoarthritis joints exerts an inflammation modulatory effect. 

The subjective findings indicate participants felt more confident about their knees when performing daily activities. Majority of objective outcomes were not statistically significant, but these measurements indicated an improving trend, although not as strong as the subjective findings. This may largely be attributed to the type and extent of knee damage already present in participants. The cohort primarily consisted of participants from an average adult population between the ages of 40–60 with occupations that varied from highly sedentary work to manual labor work such as construction and farming. Participants included patients with either RA or OA. Further, the present study has a small size and limited statistical power. Additionally, AuNP at micro-dosage 0.34 mg daily might not be the optimal dosage to achieve the best outcomes, and therefore a dose-dependent clinical study could be valuable and informative.

Other limitations of the present study were unique given the year the study was performed. Higher-than-normal attrition and skipped appointment rates were largely due to the COVID-19 pandemic. This resulted in varying amounts of completed assessments during each time interval (T0, T1, T2, and T3). It also interrupted daily dosing of the supplement for four participants, as they experienced nausea and vomiting from the COVID virus. 

Thus, it is recommended that a study be repeated with a larger cohort. By adding more participants, the data will obtain higher statistical power, giving more reliable results with statistical significance. It might also be worthwhile to conduct a repeat study only in RA or OA patients, instead of combining both types. 

This study opens the gateway for using AuNP supplement for joint health and function. Previous studies have focused on AuNP in relatively large doses treating RA, more specifically, the pain and swelling associated with RA. However, smaller micro-doses of elemental gold used in supplements have not been explored, nor has their benefit in the sports and recreation field, or in non-arthritic joints, been researched to its full extent. This study could provide a foundation for the use of micro-dose AuNP for joint health and function. It might be possible that AuNP supplement can be used to help decrease discomfort felt from soreness after strenuous exercise and may play a role in athletic performance.

Lastly, since AuNP supplement does not affect the gastrointestinal environment, unlike more commonly used NSAIDs such as ibuprofen, it should be studied for benefits in those that suffer from acute joint injuries, such as fractures, dislocations, etc., as the supplement might serve as an alternative to NSAIDs. Further studies should be undertaken to compare the effectiveness of NSAIDs versus AuNPs.

## 6. Conclusions

The study found that the participants’ perceptions of their knees significantly improved after taking AuNP supplement. The study showed a statistically significant increase in KOOS Survey scores for pain, knee symptoms, activities of daily living, sports and recreational activities, and quality of life after the participants’ first two months AuNP supplement. Although the majority of objective measures were not statistically significant, they trended towards improvement, supporting effectiveness of the supplement in improving knee joint health and function. This study makes way for the use of AuNP in both clinical and daily settings to improve joint health and function for both average and athletic users. 

The study is limited by its small sample size and low dosage, and thus lack of statistical power. A dose-dependent study with bigger study size can be valuable and informative. 

## Figures and Tables

**Table 1:** KOOS Scores Changes for First Use of Supplement Cohort. The Supplement Group’s T0–T1 and the Placebo Group’s T2–T3 phase intervals scores were combined to form the First Use of Supplement Cohort (*n* = 51). For each of the five categories, the changes were statistically significant (*p* < 0.01).

	PAIN	SYMPTOMS	ADL	SPORTS & REC	QOL
Average Score Change for First Use of Supplement (Standard Deviation)	9.96 (14.50)	6.32 (14.80)	10.64 (16.62)	10.69 (18.17)	12.24 (16.58)
95% Confidence Interval; *p*	5.80, 14.13; *p* < 0.01	2.07, 10.57; *p* < 0.01	5.87, 15.41; *p* < 0.01	5.36, 16.03; *p* < 0.01	7.48, 17.01; *p* < 0.01
Total Number of Improved Participants During First Use Supplement (%)	37 (75.5%)	28 (57.1%)	35 (71.4%)	31 (66%)	33 (67.3%)
Coefficient of Variation (CV)	1.46	2.34	1.56	1.70	1.35

**Table 2:** Best “Effect” Estimate of Pain with ROM. Fixed effects from multilevel model of pain with ROM at times T0 vs. T1 (Phase 1).

T0 vs. T1	Estimate	Standard Deviation	z Value	Pr(>|z|)
(Intercept)	−0.895	0.561	−1.596	0.111
Group Supplement	0.822	0.766	1.073	0.283
Time T1	−0.689	0.596	−1.156	0.248
Group Supplement × Time T1	−0.767	0.845	−0.907	0.364

**Table 3:** Best “Effect” Estimate of Effusion. Fixed effects from multilevel model of effusion at times T0 vs. T1.

T0 vs. T1	Estimate	Standard Deviation	z Value	Pr(>|z|)
(Intercept)	−0.718	0.520	−1.382	0.167
Group Supplement	−0.154	0.710	−0.217	0.828
Time T1	−1.022	0.607	−1.684	0.092
Group Supplement × Time T1	0.710	0.820	0.866	0.387

**Table 4:** Best “Effect” Estimate of Knee Grinding. Fixed effects from multilevel model of knee grinding at times T0 vs. T1.

T0 vs. T1	Estimate	Standard Deviation	z Value	Pr(>|z|)
(Intercept)	2.565	2.426	1.057	0.290
Group Supplement	0.321	1.629	0.197	0.844
Time T1	−0.444	0.959	−0.463	0.643
Group Supplement × Time T1	−0.404	1.337	−0.303	0.762

**Table 5:** Best “Effect” Estimate of Patellar Tracking. Fixed effects from multilevel model of patellar tracking at times T0 vs. T1.

T0 vs. T1	Estimate	Standard Deviation	z Value	Pr(>|z|)
(Intercept)	0.399	0.646	0.618	0.537
Group Supplement	−1.086	0.952	−1.141	0.254
Time T1	−2.243	0.931	−2.409	0.016
Group Supplement × Time T1	1.679	1.154	1.454	0.146

## Data Availability

The data presented in this study are available on request from the corresponding author.
